# Development and validation of an ultrasound-based radiomics nomogram for predicting the luminal from non-luminal type in patients with breast carcinoma

**DOI:** 10.3389/fonc.2022.993466

**Published:** 2022-11-28

**Authors:** Jiangfeng Wu, Lifang Ge, Yun Jin, Yunlai Wang, Liyan Hu, Dong Xu, Zhengping Wang

**Affiliations:** ^1^ Department of Ultrasound, Dongyang People’s Hospital, Dongyang, Zhejiang, China; ^2^ Department of Diagnostic Ultrasound Imaging and Interventional Therapy, The Cancer Hospital of the University of Chinese Academy of Sciences (Zhejiang Cancer Hospital), Hangzhou, China; ^3^ Institute of Basic Medicine and Cancer (IBMC), Chinese Academy of Sciences, Hangzhou, China

**Keywords:** ultrasound, breast carcinoma, radiomics, non-luminal type, luminal type

## Abstract

**Introduction:**

The molecular subtype plays a significant role in breast carcinoma (BC), which is the main indicator to guide treatment and is closely associated with prognosis. The aim of this study was to investigate the feasibility and efficacy of an ultrasound-based radiomics nomogram in preoperatively discriminating the luminal from non-luminal type in patients with BC.

**Methods:**

A total of 264 BC patients who underwent routine ultrasound examination were enrolled in this study, of which 184 patients belonged to the training set and 80 patients to the test set. Breast tumors were delineated manually on the ultrasound images and then radiomics features were extracted. In the training set, the T test and least absolute shrinkage and selection operator (LASSO) were used for selecting features, and the radiomics score (Rad-score) for each patient was calculated. Based on the clinical risk features, Rad-score, and combined clinical risk features and Rad-score, three models were established, respectively. The performances of the models were validated with receiver operator characteristic (ROC) curve and decision curve analysis.

**Results:**

In all, 788 radiomics features per case were obtained from the ultrasound images. Through radiomics feature selection, 11 features were selected to constitute the Rad-score. The area under the ROC curve (AUC) of the Rad-score for predicting the luminal type was 0.828 in the training set and 0.786 in the test set. The nomogram comprising the Rad-score and US-reported tumor size showed AUCs of the training and test sets were 0.832 and 0.767, respectively, which were significantly higher than the AUCs of the clinical model in the training and test sets (0.691 and 0.526, respectively). However, there was no significant difference in predictive performance between the Rad-score and nomogram.

**Conclusion:**

Both the Rad-score and nomogram can be applied as useful, noninvasive tools for preoperatively discriminating the luminal from non-luminal type in patients with BC. Furthermore, this study might provide a novel technique to evaluate molecular subtypes of BC.

## Introduction

Breast carcinoma (BC) is among the tumors with the highest morbidity and mortality in women, which accounts for one in four cancer cases and for one in six cancer deaths in the great majority of countries (1). BC is a heterogeneous disease with different clinical characteristics, clinical behaviors, and treatment response profiles (2). There are four major subtypes of breast carcinoma based on estrogen receptor (ER), progesterone receptor (PR) and human epidermal growth factor receptor 2 (HER2) status. These intrinsic molecular subtypes are defined as: luminal A and B, HER2-enriched, and basal-like subtypes. Luminal A and B are luminal type, while HER2-enriched and basal-like are non-luminal type (3).

Luminal type shows better prognosis and response to hormone receptor-targeted therapies (4), while non-luminal type is more aggressive, has poorer outcomes than luminal type, and reveals a higher rate of locoregional recurrence and lower survival rate after distant metastasis (5–7). Since the prognosis of BC differs according to the molecular subtypes, guidelines recommend for immunohistochemistry (IHC) assessment of the molecular subtypes of BC during initial diagnosis (8, 9).

Currently, the preoperative assessment of the molecular subtypes of BC commonly relies on IHC results after core needle biopsy. Whereas, biopsy is invasive and vulnerable to sampling error that might negatively affect the treatment decision and increase health care costs of BC diagnosis when repeated biopsy is required (10, 11). Moreover, the limited biopsy tissue makes it difficult to fully evaluate the heterogeneity within the tumor (12). Therefore, a noninvasive approach that could precisely predict the molecular subtypes of BC, would be high valuable in the early management of patients with BC.

Radiomics is an innovative tool that usually extracts a large number of quantitative features from medical images by using mathematical algorithms, and the quantitative extracted features can represent the shape, intensity, and texture of tumors. It assumes that the quantitative features may be the reflection of mechanisms occurring at a genetic and molecular level, and relevant to tumor behavior or patient’s prognosis (13, 14). Radiomics features have been employed to noninvasively evaluate intratumoral heterogeneity. In recent years, studies have found that radiomics analysis can be utilized to determine sentinel lymph node metastasis (15, 16), distinguish between the benign and malignant breast tumor (17, 18), identify the molecular subtype (19), and evaluate the response to neoadjuvant chemotherapy (20).

Several studies have demonstrated that radiomics features based on computed tomography (CT) or magnetic resonance imaging (MRI) have the ability to discriminate the luminal from non-luminal BC. Wang and colleagues (21) established a radiomics model based on chest CT to distinguish the luminal from non-luminal type and achieved area under the curve (AUC) values of 0.842 in the training set and 0.757 in the test set, demonstrating that chest CT radiomics may provide a new concept for the identification of breast cancer molecular subtypes. The results of a former study by Leithner et al. (22) indicated the potential of radiomics signatures from multiparametric MRI to allow the separation of the hormone receptor-positive and hormone receptor-negative BC. However, no study based on ultrasound radiomics has been published on distinguishing between the luminal and non-luminal BC. Furthermore, compared to CT and MRI, ultrasound regarded as a nonradioactive, available, and low-cost tool is commonly adopted for breast tumor screening and diagnosis.

Therefore, based on the above background, we studied whether ultrasound radiomics features could be adopted as a predictive biomarker for discriminating the luminal from non-luminal type in patients with BC, and the purpose of this study was to develop and validate an ultrasound-based radiomics nomogram by integrating the clinical risk factors and radiomics score (Rad-score) to preoperatively predict the luminal BC.

## Materials and methods

The study was approved by our Institutional Ethics Committee and performed on the basis of the Helsinki Declaration, and patient informed consent requirement was waived due to the retrospective nature of this study.

### Patient cohort

Patients who were diagnosed with BC and had preoperative breast ultrasound between March 2019 and July 2021 were included.

The inclusion criteria were (a) patients diagnosed as BC by surgical or biopsy pathology; (b) lesions presenting as mass (facilitating the subsequent segmentation of breast tumors); (c) ultrasound examinations were performed within two weeks before surgery; and (d) the presence of single malignant tumor.

The exclusion criteria were (a) patients with ductal carcinoma *in situ* confirmed by histopathology; (b) images with prominent artifacts; (c) tumors larger than 50 mm in diameter (incompletely displayed in a single plane); (d) patients who underwent biopsy or chemoradiotherapy before ultrasound examination; and (e) patients with missing clinical characteristics and/or postoperative IHC.

**Figure 1:**
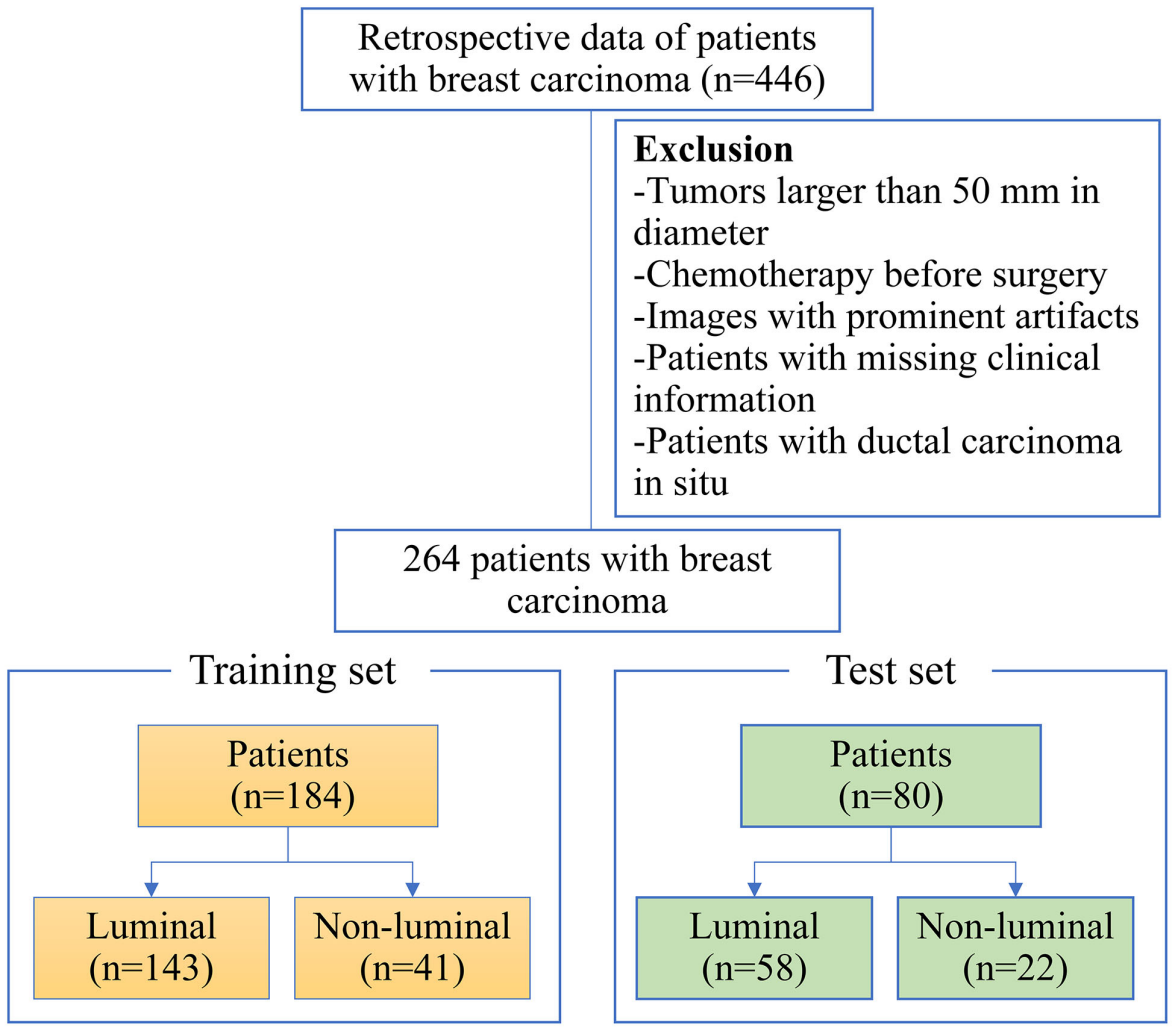
The patient enrollment process for this study.

On the basis of inclusion criteria, 446 patients were reviewed. Applying our exclusion criteria, a total of 264 patients were therefore included finally. The flowchart of patient selection process was revealed in **Figure 1**. The 264 patients were randomly divided into the training and test sets according to the ratio of 7:3. we analyzed 184 patients in the training set and 80 patients in the test set. The training set included 143 and 41 patients with luminal and non-luminal type, respectively, while the test set included 58 and 22 patients with luminal and non-luminal type, respectively.

### Pathological assessment

The surgical or ultrasound-guided needle biopsy pathology was obtained for the diagnosis of the target breast tumor. If the tumor was malignant, IHC analysis was further performed. IHC analyses were carried out to detect the expression levels of ER, PR, HER2, and Ki-67 in each patient with BC. The status of ER and PR was considered as positive, if greater than 1% of tumor cells revealing positively stained nuclei (23). For HER2 status identification, a HER2 staining intensity score of 3+ was regarded as positive, while a score of 0 or 1+ was considered as negative. A HER2 staining intensity score of 2+, with confirmation of gene amplification by fluorescence *in situ* hybridization, was also regarded as positive (3). For Ki-67 status, tumors with greater than 14% positive nuclei were considered as high expression, while other cases were considered as low expression (3).

### Clinical and pathological characteristics

Clinical data, such as age, US-reported tumor size, tumor location, ultrasound equipment, ultrasound-reported lymph node metastasis, ER status, PR status, HER2 status, Ki-67 level, and pathology-reported lymph node metastasis were recorded both in the training and test sets, and the molecular subtypes of breast carcinoma were calculated by the status of ER, PR and HER2.

### Image acquisition and segmentation

All breast ultrasound examinations were performed by two sonographers (JW and YW with more than 5 years’ experience in breast ultrasound imaging) by using LOGIQ E9 ultrasound system with a 6-15L linear array probe and Siemens Acuson S2000 with a 6-18L linear array probe with radial, transverse, and longitudinal scans on both breasts. Ultrasound was further utilized to scan breast tumor from multiple angles and sections to absorb the overall information. In addition, we carefully observed the shape, size, blood supply and echo of the tumor. The scan parameters were consistent among patients: image depth was about 4.0 cm; gain was about 50%; and focus paralleled to the tumor. The image of the largest section of the breast tumor with the clearest imaging was saved as the format of Digital Imaging and Communications in Medicine to maximize the preservation of the image information. A two dimensional region of interest (ROI) that covered the whole lesion was manually delineated on the selected largest section of the breast tumor by using ITK-SNAP software (open source software; http://www.itk-snap.org). This was performed independently by an experienced sonographer (YJ with more than 5 years’ experience in breast ultrasound imaging) blinded to patients’ postoperative IHC.

### Feature extraction

A total of 788 quantitative radiomics features were then extracted from each ROI using the “pyradiomics” package of Python (version 3.7.11). These ultrasound radiomics features were divided into four categories including shape, statistics, texture and wavelet features: 14 two dimension shape-based features, 18 first-order statistics features, 22 gray-level co-occurrence matrix (GLCM) features, 16 gray-level run length matrix (GLRLM) features, 16 gray-level size zone matrix (GLSZM) features, 14 gray-level dependence matrix (GLDM) features, and 688 features derived from first-order, GLCM, GLRLM, GLSZM and GLDM features using wavelet filter images (24).

### Evaluation of interclass correlation coefficient

To ensure the reproducibility and accuracy, 50 patients were randomly selected for a reproducibility analysis by using the interclass correlation coefficient (ICC). Two sonographers (YJ and YW with more than 5 years’ experience in breast ultrasound imaging) drew ROIs on the same ultrasound images from the 50 selected patients and extracted the radiomics features. An ICC greater than 0.70 suggested a good agreement.

### Radiomics feature selection

The radiomics features data were normalized with z score normalization in the training and test sets to ensure that the scale of feature value was uniform and improve the comparability between features. A two-step feature selection procedure was designed for mining the valuable predictive features in the training set. First, a T test was implemented to remove the features that showed no significant difference between the luminal and non-luminal BC. Next, the least absolute shrinkage and selection operator (LASSO) with ten-fold cross validation was adopted to further select the features through regularization by optimizing the hyperparameter (Lambda). An optimum Lambda was tuned to achieve minimum mean square error. Coefficients of some candidate features were compressed to zero at the optimum Lambda, and features with nonzero coefficients were retained.

### Construction and validation of prediction models

**Figure 2:**
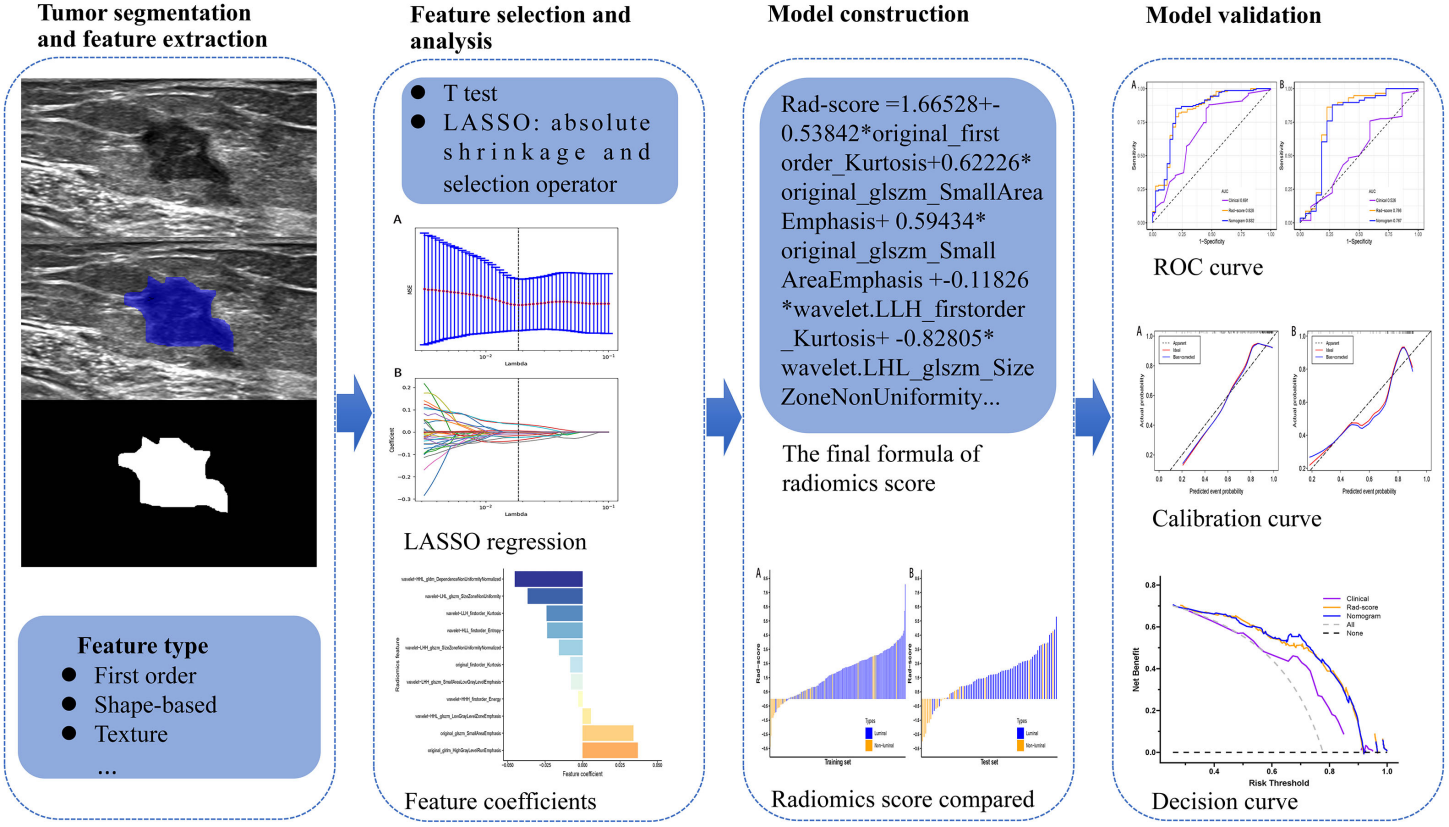
Flowchart of the processing step using the radiomics method for predicting the luminal BC.

After feature selection, The Rad-score of each patient with BC was calculated with a linear combination of the final selection of features weighted by logistic regression algorithm. Meanwhile, logistic regression model based on Rad-score was developed for identifying the luminal type. Furthermore, clinical features that showed a statistical difference between the luminal and non-luminal type in the training set was adopted to develop the clinical model. At the same time, the nomogram based on the clinical features and Rad-score would be constructed. The sensitivity, specificity, positive predictive value (PPV), negative predictive value (NPV), accuracy, and AUC were adopted to quantify the predictive performance of the models. To verify the robustness of the nomogram, the calibration curve (25) was plotted. Furthermore, decision curve analysis (DCA) (26) was utilized to select the model that maximized patient benefits. The flowchart of this research is shown in **Figure 2**.

### Statistical analysis

All statistical analyses were performed with the R software (version 3.5.1; www.r-project.org). The continuous variables with normal distribution were shown as mean ± standard deviation (SD), and non-normal were shown as the median. For continuous clinical or pathological variables, independent sample T test or Mann-Whitney U test was adopted to identify differences between the training and test sets. The Fisher’s exact test or Chi-square test was used for comparing categorical variables. For all statistical tests, differences were considered significant at p < 0.05.

## Results

### Clinical and pathological characteristics

The clinical and pathological characteristics of the training and test sets were compared, and there was no statistically significant difference found (p > 0.05) (**Table 1**). This suggested that the training and test sets were comparable in these clinicopathological features.

**Table 1:** The baseline characteristics of the enrolled patients in the training and test sets.

Characteristic	Total set (n = 264)	Training set (n = 184)	Test set (n = 80)	*p*-value
**Age** (year, mean ± SD)	53.41 ± 11.20	52.95 ± 11.61	54.46 ± 10.20	0.289
**US-reported tumor size** (mm, mean ± SD)	25.16 ± 11.36	24.45 ± 11.05	26.80 ± 11.96	0.136
**Location of tumor**				0.693
Right lobe	142	97	45	
Left lobe	122	87	35	
**Molecular type**				0.641
Luminal A	138	100	38	
Luminal B	62	42	20	
HER2-enriched	28	17	11	
Triple negative	36	25	11	
**ER**				0.384
Positive	199	142	57	
Negative	65	42	23	
**PR**				0.741
Positive	164	116	48	
Negative	100	68	32	
**HER2**				0.110
Positive	67	41	26	
Negative	197	143	54	
**Histologic type**				0.312
Invasive ductal	228	162	66	
Other	36	22	14	
**Ultrasound equipment**				0.645
Siemens Acuson S2000	214	151	63	
LOGIQ E9	50	33	17	
**US-reported LN**				0.097
Metastasis positive	115	74	41	
Metastasis negative	149	110	39	
**Pathology-reported LN**				0.677
Metastasis positive	155	106	49	
Metastasis negative	109	78	31	
**Ki-67** (%, mean ± SD)	28.50 ± 22.36	28.32 ± 22.93	28.91 ± 21.13	0.837
**Radiomics score** (median, IQR)	1.767 (0.566, 2.821)	1.914 (0.562, 2.963)	1.679 (0.616, 2.629)	0.352

ER, estrogen receptor; PR, progesterone receptor; HER2, human epidermal growth factor receptor 2; SD, standard deviation; IQR, interquartile range; LN, lymph node; US, ultrasound.

### Radiomics feature extraction and selection

A total of 788 ultrasound radiomics features were extracted from the ultrasound images of each patient. The details about ultrasound radiomics extraction settings are available in Supplementary Material Data S1. All the 788 features showed an interclass correlation coefficient of greater than 0.70. After validation of interobserver reproducibility, the following analyses were based on the radiomics features extracted by sonographer YJ.

**Figure 3:**
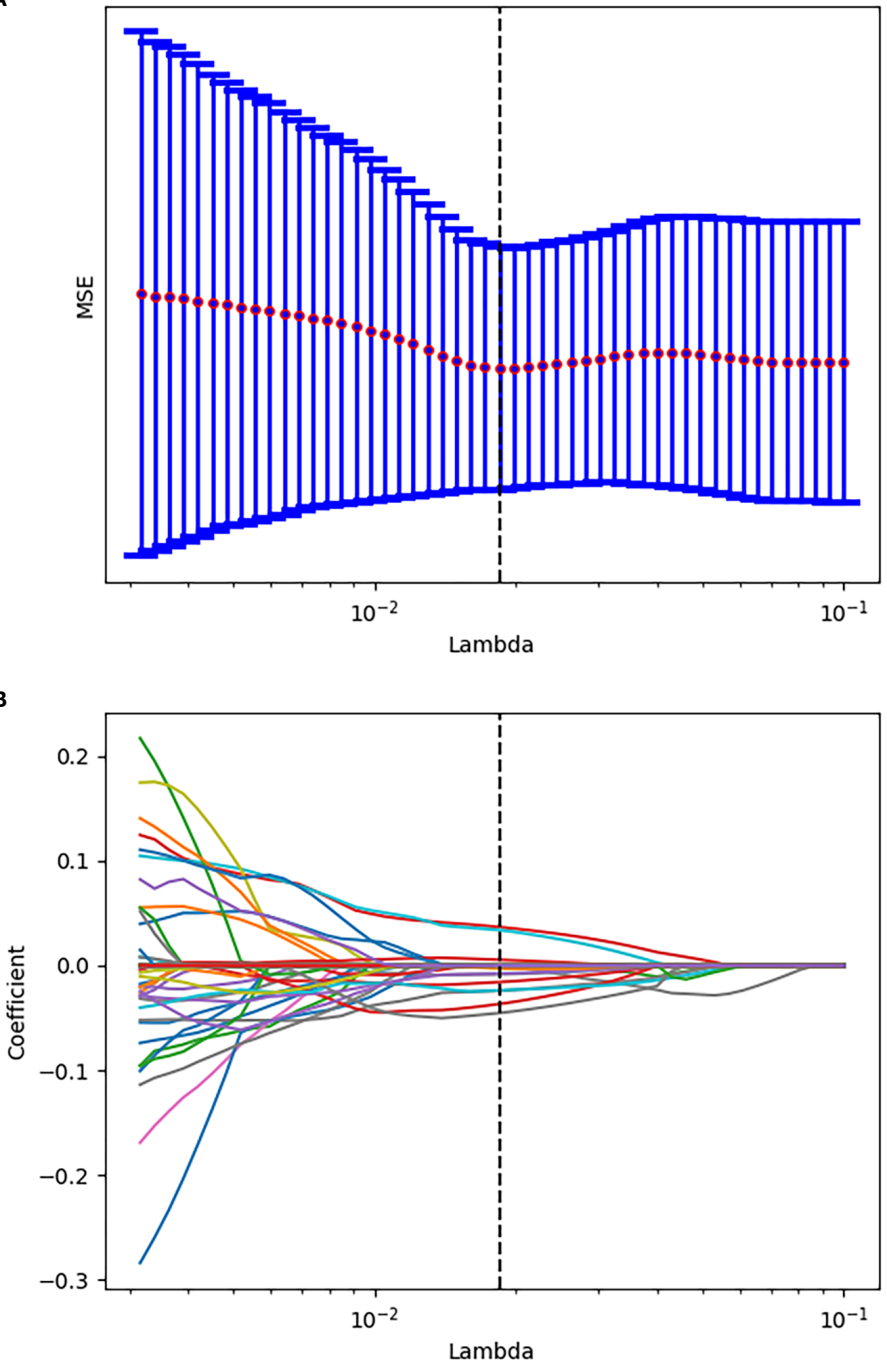
Tuning parameter selection using the LASSO regression in the training set. **(A)** The optimal penalization coefficient lambda was generated in the LASSO *via* 10-fold cross validation. The lambda value of the minimum mean square error for the training set was given; **(B)** LASSO coefficient profiles of the radiomics features.

**Figure 4:**
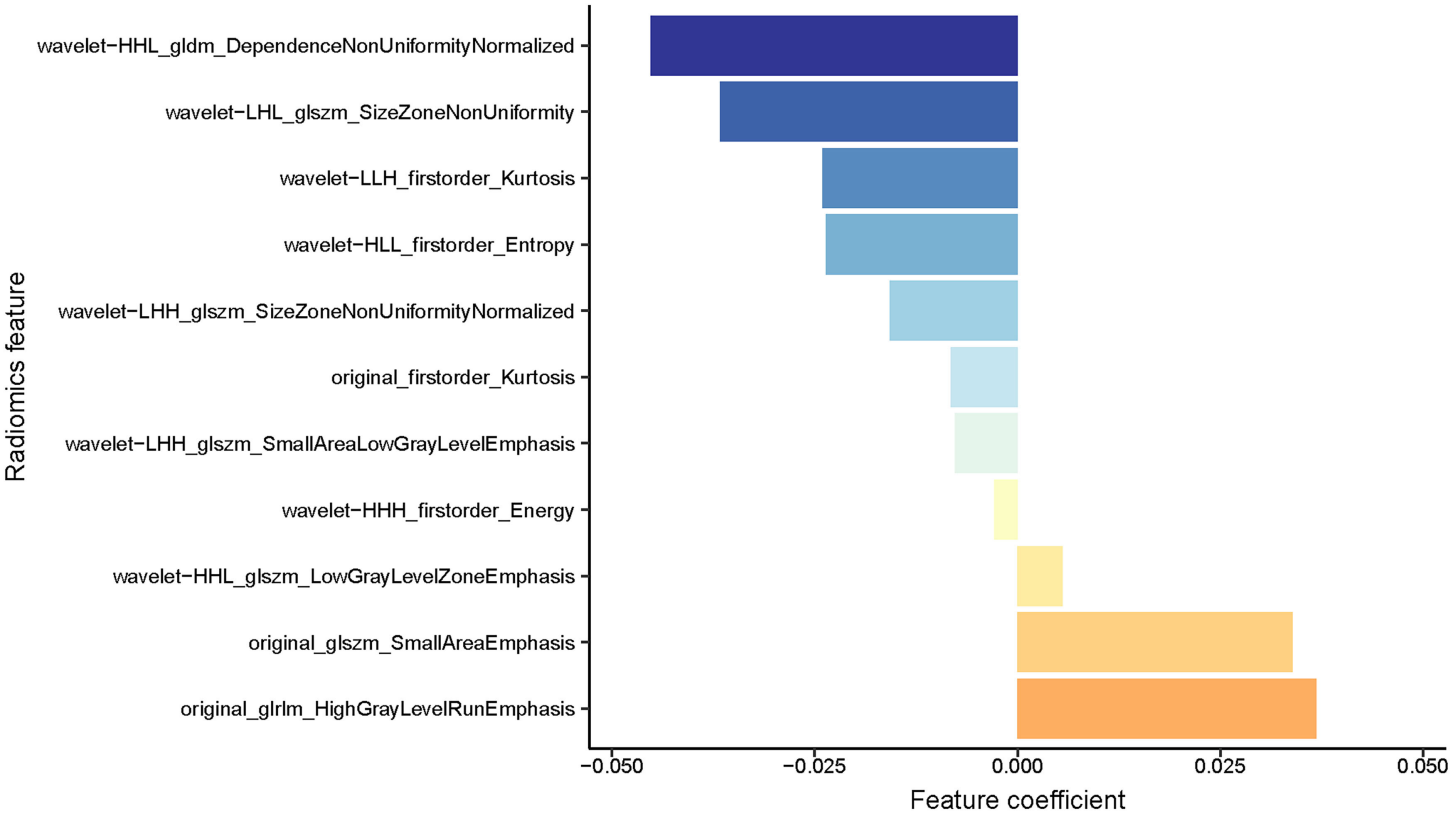
A coefficient profile plot of the 11 selected radiomics features based on the LASSO algorithm was drawn.

**Figure 5:**
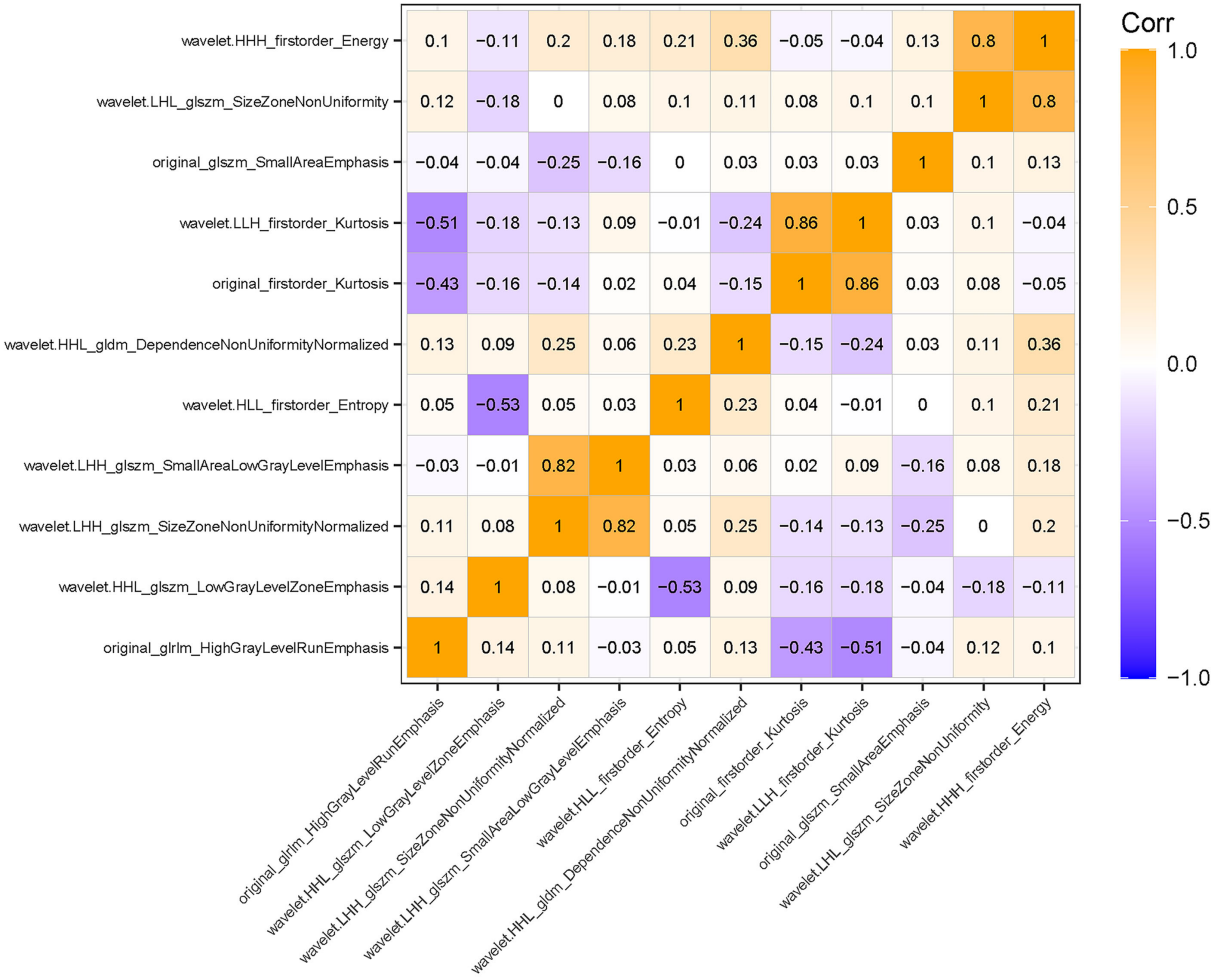
Pearson correlation coefficient heatmap of the selected features on differentiation between the luminal and non-luminal BC. Orange color denotes a positive correlation, blue color denotes a negative correlation, and the shade of the color indicates the correlation degree.

After evaluating the differences of radiomics features by using the T test, 135 radiomics features were used for the subsequent analysis. Then, the optimum Lambda (Lambda = 0.018420699693267165) was determined for the LASSO regression, and 11 radiomics features with nonzero coefficients were selected to differentiate the luminal from non-luminal BC (**Figure 3**). Detailed information on the luminal type related features is revealed in **Table 2** and the nonzero coefficients of the selected features based on the LASSO regression are shown in **Figure 4**. Moreover, the Pearson correlation coefficient between any pair of selected features was computed, and the Pearson correlation coefficient matrix heatmap is revealed in **Figure 5**.

**Table 2:** List of the selected features with nonzero coefficients.

Image type	Feature class	Feature name	Coefficient
original	firstorder	Kurtosis	-0.008190
original	glrlm	HighGrayLevelRunEmphasis	0.036799
original	glszm	SmallAreaEmphasis	0.033869
wavelet-LLH	firstorder	Kurtosis	-0.024010
wavelet-LHL	glszm	SizeZoneNonUniformity	-0.036624
wavelet-LHH	glszm	SizeZoneNonUniformityNormalized	-0.015742
wavelet-LHH	glszm	SmallAreaLowGrayLevelEmphasis	-0.007676
wavelet-HLL	firstorder	Entropy	-0.023619
wavelet-HHL	glszm	LowGrayLevelZoneEmphasis	0.005541
wavelet-HHL	gldm	DependenceNonUniformityNormalized	-0.045262
wavelet-HHH	firstorder	Energy	-0.002819

### Prediction based on Rad-score

**Figure 6:**
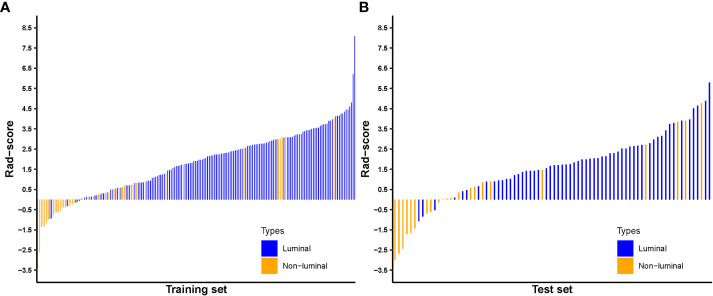
Radiomics score for each breast carcinoma patient in the training **(A)** and test sets **(B)**.

**Figure 7:**
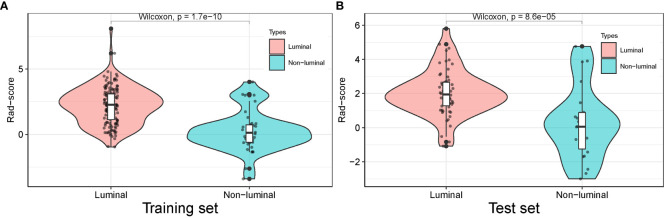
Distribution of radiomics score value of the luminal and non-luminal BC in the training **(A)** and test sets **(B)**.

The Rad-score for each patient in the training and test sets was calculated with selected features by using the logistic regression algorithm for further analysis and revealed in **Figure 6**. The corresponding fitting formula is listed in Supplementary Material Data S2. In the training set, the medians of Rad-score were statistical difference between the luminal and non-luminal type (2.267 vs. 0.133, p < 0.001), and the same results were achieved in the test set (1.948 vs. 0.054, p < 0.001) (**Figure 7**, **Table 3**).

**Table 3:** Rad-score for the training and test sets.

Rad-score	Luminal type (median, IQR)	Non-luminal type (median, IQR)	*p*-value
Training set	2.267 (1.139, 3.100)	0.133 (-0.598, 0.734)	<0.001
Test set	1.948 (1.288, 2.667)	0.054 (-1.245, 0.885)	<0.001

IQR, interquartile range.

The predictive performance of the Rad-score was well, with AUC values of 0.828 (95% confidence interval (CI), 0.747-0.911) in the training set and 0.786 (95% CI, 0.635-0.924) in the test set. Furthermore, the sensitivity, specificity, accuracy, positive predictive value (PPV) and negative predictive value (NPV) were 81.82%, 78.05%, 80.98%, 92.86% and 55.17% in the training set, and 86.21%, 77.27%, 83.75%, 90.91% and 68.00% in the test set.

### Prediction based on radiomics nomogram

**Figure 8:**
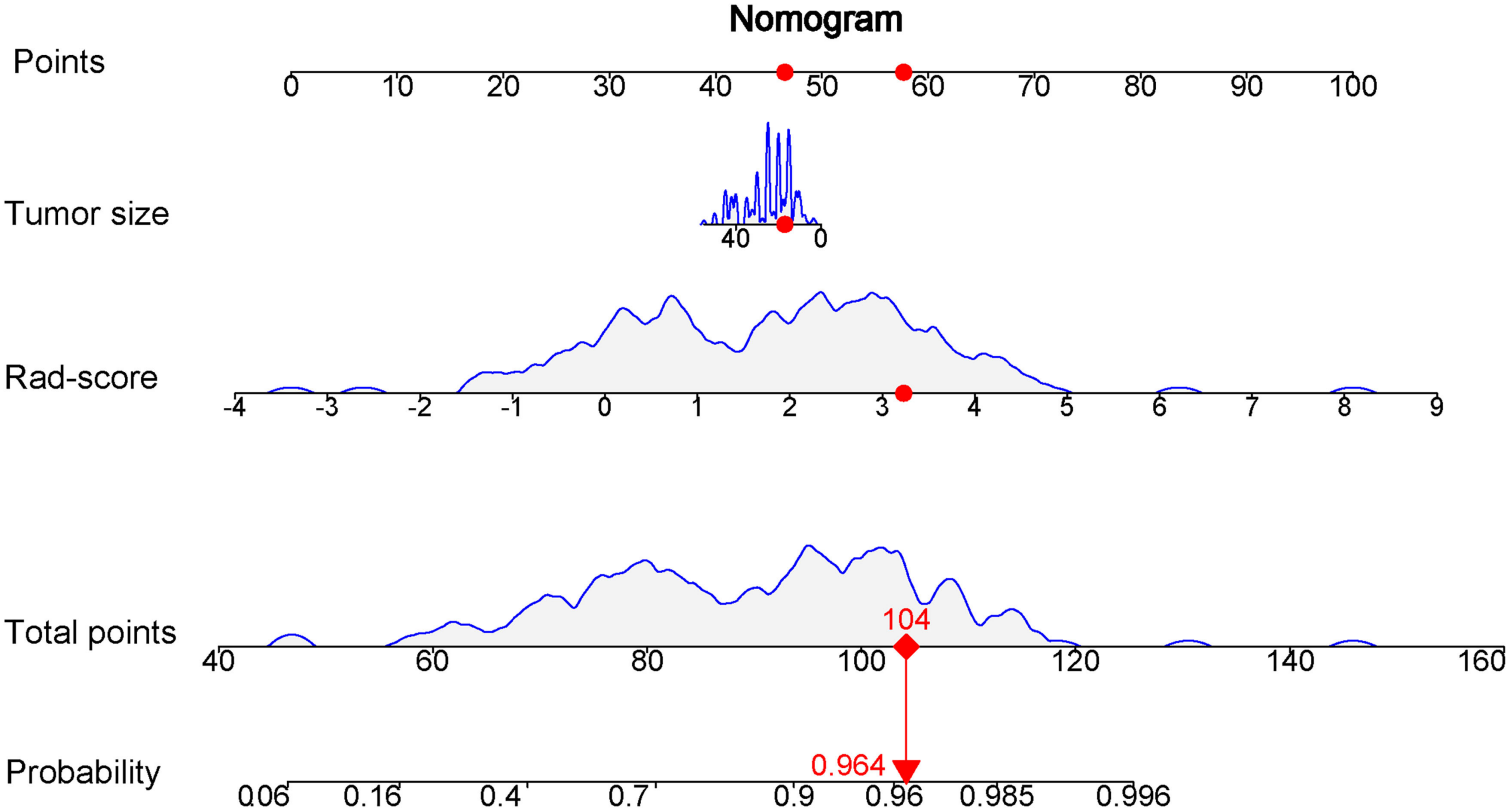
Nomogram based on the combination of the US-reported tumor size and Rad-score was developed using logistic regression analysis. If a patient with the radiomics score of 3.231 and US-reported tumor size of 17 mm, and then the probability of the luminal breast carcinoma is 0.964 (red numbers).

Comparison of the clinical features between the luminal and non-luminal BC in the training set was performed. US-reported tumor size (p < 0.001) and Rad-score (p < 0.001) were the significant factors to distinguish the luminal from non-luminal BC. Other clinical features such as age, tumor location, ultrasound equipment and ultrasound-reported lymph node status were not identified as potential factors for predicting the luminal type (**Table 4**). Therefore, the Rad-score and US-reported tumor size were incorporated to develop the radiomics-based nomogram (**Figure 8**). The sensitivity, specificity, accuracy, PPV, NPV and AUC value for the nomogram were 85.31%, 80.49%, 84.24%, 93.85%, 61.11% and 0.832 (95% CI, 0.751-0.915) in the training set, and 87.93%, 72.73%, 83.75%, 89.47%, 69.57% and 0.767 (95% CI, 0.614-0.906) in the test set, respectively.

**Table 4:** Comparison of the clinical features between the luminal and non-luminal BC in the training set.

	Training set (n = 184)		
Clinical feature	Luminal (n = 143)	Non-luminal (n = 41)	*p*-value
Age (year, mean ± SD)	53.13 ± 11.25	52.32 ± 12.92	0.718
Location			0.453
Right	78	19	
Left	65	22	
US-reported tumor size (mm, mean ± SD)	22.48 ± 10.01	31.32 ± 11.86	**< 0.001**
US equipment			0.946
Siemens Acuson S2000	118	33	
LOGIQ E9	25	8	
US-reported LN			0.147
Metastasis positive	53	21	
Metastasis negative	90	20	
Rad-score (median, IQR)	2.267 (1.139, 3.100)	0.133 (-0.598, 0.734)	**< 0.001**

SD, standard deviation; LN, lymph node; US, ultrasound; IQR, interquartile range. Bold mean statistic difference.

Ten-fold cross validation was applied to the nomogram model, which yielded median AUC values of 0.825 and 0.765 for the separation of the luminal from non-luminal BC in the training and test sets, with median accuracies of 83.36% in the training set and 80.05% in the test set.

### Prediction based on clinical risk factors

At the same time, the prediction model on the basis of US-reported tumor size was constructed. The sensitivity, specificity, accuracy, PPV, NPV and AUC value for the clinical model were 88.03%, 52.38%, 79.89%,86.21%, 56.41% and 0.691 (95% CI, 0.610-0.803) in the training set, and 75.86%, 40.91%, 66.25%, 77.19%, 39.13% and 0.526 (95% CI, 0.377-0.675) in the test set, respectively.

**Figure 9:**
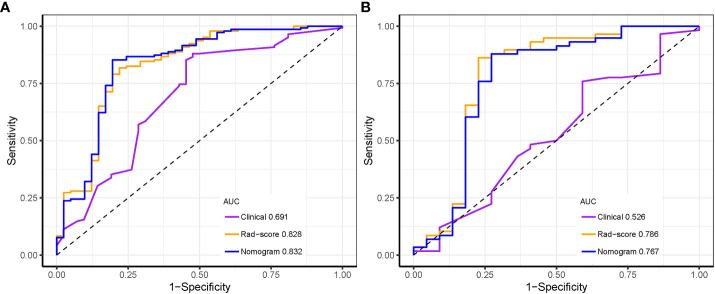
Receiver operating characteristic curves of the three models distinguishing the luminal from non-luminal type in the training **(A)** and test sets **(B)**.

The predictive values of three prediction models, including Rad-score, radiomics nomogram and clinical feature, were compared. In the training set, the AUC value of the nomogram was higher than that of the clinical model alone (AUC, 0.832 vs. 0.691; DeLong test, p < 0.001), and there was no statistical difference between the nomogram and Rad-score (AUC, 0.832 vs. 0.828; DeLong test, p = 0.107). In the test set, the AUC value of the nomogram was higher than that of the clinical model alone (AUC, 0.767 vs. 0.526; DeLong test, p < 0.001), and there was no statistical difference between the nomogram and Rad-score (AUC, 0.767 vs. 0.786; DeLong test, p = 0.286). The discrimination performance for each model is summarized in **Table 5**. Receiver operator characteristic (ROC) curves of the three models to predict the luminal type are shown in **Figure 9**.

**Table 5:** Performances of the models in the prediction of the luminal breast carcinoma.

Model	Cohort	AUC (95% CI)	SEN (%)	SPE (%)	ACC (%)	PPV (%)	NPV (%)
Clinical	Training	0.691 (0.610-0.803)	88.03%	52.38%	79.89%	86.21%	56.41%
	Test	0.526 (0.377-0.675)	75.86%	40.91%	66.25%	77.19%	39.13%
Rad-score	Training	0.828 (0.747-0.911)	81.82%	78.05%	80.98%	92.86%	55.17%
	Test	0.786 (0.635-0.924)	86.21%	77.27%	83.75%	90.91%	68.00%
Nomogram	Training	0.832 (0.751-0.915)	85.31%	80.49%	84.24%	93.85%	61.11%
	Test	0.767 (0.614-0.906)	87.93%	72.73%	83.75%	89.47%	69.57%

AUC, area under the curve; SEN, sensitivity; SPE, specificity; ACC, accuracy; PPV, positive predictive value; NPV, negative predictive value; Rad-score, radiomics score.

### Clinical application of prediction models

**Figure 10:**
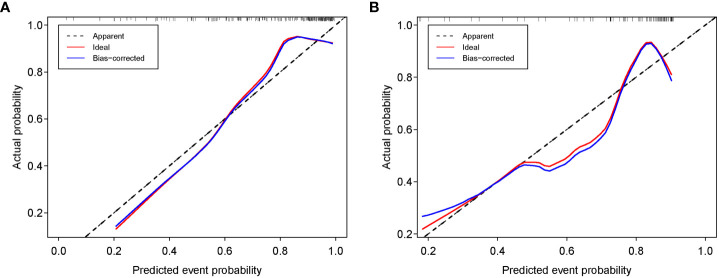
Calibration curves of the nomogram in the training **(A)** and test sets **(B)**.

The calibration curve for the nomogram was tested using Hosmer-Lemeshow test, and yielded nonsignificant results due to both p values > 0.05 in the training and test sets, showing good agreements between the observed and predicted results. (**Figure 10**).

**Figure 11:**
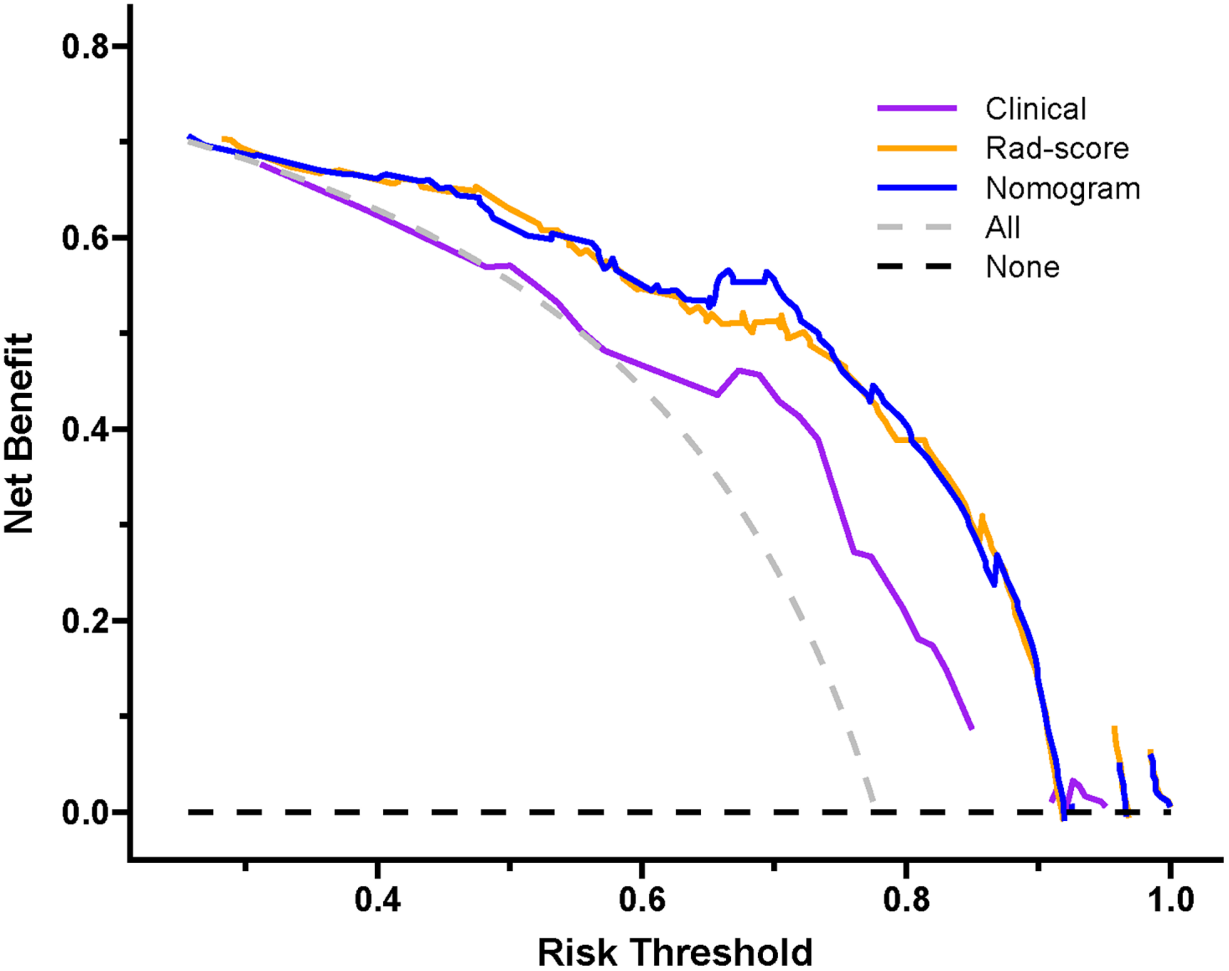
Decision curve of the nomogram. If the risk threshold is less than 92.1%, the nomogram model will obtain more benefit than all treatment (assuming all breast carcinoma patients were luminal type) or no treatment (assuming all breast carcinoma patients were non-luminal type).

Decision curve analysis of the three prediction models are revealed in **Figure 11**. The y-axis measures the net benefit. The grey line represents the assumption that all lesions were luminal type. The black line represents the assumption that all lesions were non-luminal type. If the threshold probability was less than 92.1%, using the nomogram added more benefit (blue line).

## Discussion

In the present radiomics study, feature extraction was carried out on the basis of ultrasound images, and three prediction models were established using clinical feature, Rad-score and combined clinical feature and Rad-score, respectively, for predicting the luminal BC. The results demonstrated that the Rad-score and nomogram had appreciable predictive performance and could be applied as useful methods for preoperatively differentiating between the luminal and non-luminal type in patients with BC. The consistency between the nomogram-predicted probability of luminal BC and actual results was assessed by the calibration curve. On the calibration curve in our study, both *p-*values were > 0.05 in the training and test sets, indicating that the stability of the nomogram was well. Decision curve analysis revealed that the net benefit of using the nomogram (blue curve) and Rad-score (orange curve) for predicting luminal BC was more considerable than that of the treat-all or treat none approach, indicating well clinical application of the novel models.

A number of studies have demonstrated that radiomics is regarded as an useful and noninvasive method for predicting molecular subtypes in patients with BC, most of which are on the basis of mammography and MRI imaging. Son and colleagues (27) built radiomics signatures based on synthetic mammography reconstructed from digital breast tomosynthesis to predict molecular subtypes of breast cancer. In the validation cohort, the radiomics signature yielded an AUC of 0.838, 0.556, and 0.645 for the triple-negative, HER2 and luminal subtypes, respectively. In our study, the AUC value of Rad-score predicting luminal BC was higher than theirs (0.786 vs. 0.645). This might have some relationship with low spatial resolution of mammography and limited display effect for breast tumor. A prior study by Li et al. (28) including a total of 351 patients and developing radiomics models for predicting the HER2 status preoperatively, found that the intratumoral and peritumoral radiomics scores achieved AUCs of 0.683 and 0.690 in the validation cohort, respectively. Furthermore, the combined radiomics score improved the predictive performance and yielded an AUC of 0.713. Huang and colleagues (29) reported that the multi-parametric MRI-based radiomics models could predict molecular subtypes of breast cancer. The highest performances were obtained for discriminating basal-like vs. non-basal-like (AUC, 0.965), HER2-enriched vs. non-HER2-enriched (AUC, 0.840), and hormone receptor-positive/non-HER2-enriched vs. others (AUC, 0.860) using multilayer perceptron. Compared to mammography and MRI imaging, ultrasound regarded as a nonradiative, convenient, and low-cost technology is universally used for breast tumor screening and diagnosis (30, 31). As far as we know, few studies have evaluated the feasibility of utilizing an ultrasound radiomics method in breast carcinoma to predict the luminal BC, and currently most of these focus on predicting triple-negative breast carcinoma, HER2 status, Ki-67 index, etc.

Leithner et al.’ study (22) including 91 breast cancers adopted a multi-layer perceptron feed-forward artificial neural network (MLP-ANN) to differentiate the hormone receptor-positive from hormone receptor-negative BC based on multiparametric MRI images, yielding an overall median AUC of 0.69, with median accuracies of 64.7% in the training dataset and 60.0% in the validation dataset. As compared to the above study, more samples were included in our study and applied the tenfold cross validation, the nomogram model achieved significantly higher median accuracies of 83.36% in the training set and 80.05% in the test set, with a median AUC value of 0.765 in the test set. This might lead by that the ultimate effect of the model is closely relevant to the generalization of the neural network and sample size. If the sample dataset is weakly representative, there are a number of conflictive or redundant samples, and then it is difficult for the neural network to obtain the expected result.

For the prediction of the luminal type, the AUC values of the Rad-score and nomogram were 0.786 and 0.767 in the test set. In a recent non-contrast-enhanced chest CT radiomics study (21), Wang and colleagues established forty-two models to predict the luminal type of breast cancer by the combination of six feature screening methods and seven machine learning classifiers. The final optimal model for external validation on the independent test set obtained an AUC value of 0.757. With regard to feature dimensionality reduction algorithms, they found that the overall performance of the LASSO regression was better than other dimensionality reduction methods in the field of AUC and accuracy. In our study, we also adopted LASSO algorithm for feature dimensionality reduction. In their study, the prediction model was established by seven different machine learning classifiers, among which support vector machine achieved the highest AUC value in both internal and external validations. On the contrast, in our study, models were built by using logistic regression only, and thus, further studies adopting other machine learning classifiers should be taken account in future. However, in our study, the Rad-score and nomogram all showed appreciable performance in the training and test sets. We believe that they could be utilized as a reliable technique in discriminating the luminal from non-luminal BC and may promote to assist clinicians for preoperative decision-making.

Clinical features including US-reported tumor size, age, tumor location, ultrasound equipment and ultrasound-reported lymph node metastasis were assessed in this study. Among them, US-reported tumor size (p < 0.001) showed a significantly statistical difference between the luminal and non-luminal BC in the training set. Furthermore, several previous studies have demonstrated that there was statistical difference in the terms of tumor size between the luminal and non-luminal BC (32, 33). The nomogram integrated with the US-reported tumor size showed a little higher predictive performance than that of Rad-score (AUC, 0.832 vs. 0.828) in the training set, but a little lower (AUC, 0.767 vs. 0.786) in the test set. However, no matter in the training set or in the test set, there was no statistical difference in the field of predictive performance between the nomogram and Rad-score in our study, indicating that the US-reported tumor size had limited effect to predictive performance of the nomogram. Furthermore, the US-reported tumor size could be acquired preoperatively, and then the model could be utilized for individualized prediction of the luminal type in patients with BC.

In the present study, the Rad-score was developed based on 11 luminal-related features, among which 1 first order feature, 5 glszm features, 1 glrlm feature and 1 gldm feature were included. A mix of first-order, texture and wavelet features seemed to be of importance for differentiation between the luminal and non-luminal BC, suggesting the complementary value of the combination of different radiomics features to capture different functional aspects of tumor biology. Six out of eleven radiomics features were texture features that showed the value of inter-tumor heterogeneity in predicting the gene expression (34, 35). At the same time, eight out of eleven radiomics features were wavelet features, which can also be utilized to extensively quantify heterogeneity of the tumor through different spatial scales at respective directional orientations (36). Furthermore, the first-order statistics feature such as Kurtosis appeared among the final radiomics features. Kurtosis describes the intensity value of the tumor, which is applied to many classification tasks (37, 38). Besides, radiomics features including ‘wavelet-HHL_gldm_DependenceNonUniformityNormalized’, ‘original_glrlm_HighGrayLevelRunEmphasis’, ‘wavelet-LHL_glszm_SizeZoneNonUniformity’ and ‘original_glszm_SmallAreaEmphasis’ were significantly associated with molecular subtypes of BC in ultrasound images, which had a higher proportion of the weight coefficient. However, the shape-based feature was not selected to constitute the radiomics model, indicating that the morphological characterization might be less relevant to the luminal type. Hence, we believe that tumor molecular level information can be obtained from tumor radiomics analysis and radiomics features extracted from ultrasound images of BC are available to predict molecular subtypes.

Our study had several limitations that should be taken into account. First, the retrospective nature of the analyses might have introduced selection bias. In addition, this was a single-center study with a limited number of 264 patients. Hence, future prospective studies are needed to further validate the predictive performance of the models by using a large, multi-center cohort. Second, the contour of tumor’s ROI was manually depicted by sonographers, but the judgment of tumor’s contour was easily influenced by personal subjective experience. However, we believe that this was partially solved by interobserver reproducibility assessment. Third, only gray-scale ultrasound images were adopted in our study, and in the future, we will add radiomics features of multimodal ultrasound to further studies. For example, contrast-enhanced (39) and elastography (40) ultrasound images, which may contain more radiomics features than gray-scale ultrasound images. Finally, our study was only conducted with two dimensional analysis of the largest plane of the tumor, which might not comprehensively capture the heterogeneous features of the tumor as compared to a model on the basis of three dimensional analysis. Future studies should focus on the establishment of a three-dimensional model for distinguishing the luminal from non-luminal BC.

## Conclusions

In summary, both the Rad-score and nomogram can be applied as useful, noninvasive tool for preoperatively discriminating the luminal from non-luminal type in patients with BC. Our study may provide a novel method to evaluate molecular subtypes of BC. However, further studies with a prospective design and larger population are required to validate the conclusions.

## Data availability statement

The original contributions presented in the study are included in the article/**Supplementary Material**. Further inquiries can be directed to the corresponding authors.

## Ethics statement

The studies involving human participants were reviewed and approved by the ethics committee. Written informed consent for participation was not required for this study in accordance with the national legislation and the institutional requirements.

## Author contributions

JW and LG collected the clinical and radiomics data. YW and YJ preprocessed patients’ ultrasound images and drew the ROI. JW and YJ analyzed the data and developed the prediction model. JW wrote the manuscript. ZW, DX, and LH designed the study. All authors contributed to the article and approved the submitted version.

## Funding

Jinhua Science and Technology Bureau Scientific Research Project (2022-3-019).

## Conflict of interest

The authors declare that the research was conducted in the absence of any commercial or financial relationships that could be construed as a potential conflict of interest.

## Publisher’s note

All claims expressed in this article are solely those of the authors and do not necessarily represent those of their affiliated organizations, or those of the publisher, the editors and the reviewers. Any product that may be evaluated in this article, or claim that may be made by its manufacturer, is not guaranteed or endorsed by the publisher.

## Abbreviation

BC, breast carcinoma; Rad-score, radiomics score; IHC, immunohistochemistry; MRI, magnetic resonance imaging; CT, computed tomography; CI, confidence interval; ER, estrogen receptor; PR, progesterone receptor; HER2, human epidermal growth factor receptor 2; FISH, fluorescence *in situ* hybridization; ROI, region of interest; ICC, interclass correlation coefficient; LASSO, least absolute shrinkage and selection operator; DCA, decision analysis curve; ROC, receiver operator characteristic; AUC, area under the curve; SD, standard deviation; GLCM, gray-level co-occurrence matrix; GLRLM, gray-level run length matrix; GLSZM, gray-level size zone matrix; GLDM, gray-level dependence matrix; PPV, positive predictive value; NPV, negative predictive value.
