# Supplementary material for: Development and validation of an ultrasound-based radiomics nomogram for predicting the luminal from non-luminal type in patients with breast carcinoma
Source: Front Oncol. 2022 Nov 28;12:993466. doi: 10.3389/fonc.2022.993466 (PMC9749858; doi:10.3389/fonc.2022.993466)
Supplement: Supplementary file 1 [file DataSheet_1.docx]

# This is an example of a parameters file

# It is written according to the YAML-convention (www.yaml.org) and is checked by the code for consistency.

# Three types of parameters are possible and reflected in the structure of the document:

#

# Parameter category:

# Setting Name: <value>

#

# The three parameter categories are:

# - setting: Setting to use for preprocessing and class specific settings. if no <value> is specified, the value for

# this setting is set to None.

# - featureClass: Feature class to enable, <value> is list of strings representing enabled features. If no <value> is

# specified or <value> is an empty list ('[]'), all features for this class are enabled.

# - imageType: image types to calculate features on. <value> is custom kwarg settings (dictionary). if <value> is an

# empty dictionary ('{}'), no custom settings are added for this input image.

#

# Some parameters have a limited list of possible values. Where this is the case, possible values are listed in the

# package documentation

# Settings to use, possible settings are listed in the documentation (section "Customizing the extraction").

setting:

binWidth: 25

label: 1

interpolator: 'sitkBSpline' # This is an enumerated value, here None is not allowed

resampledPixelSpacing: # This disables resampling, as it is interpreted as None, to enable it, specify spacing in x, y, z as [x, y , z]

weightingNorm: # If no value is specified, it is interpreted as None

# Image types to use: "Original" for unfiltered image, for possible filters, see documentation.

imageType:

Original: {} # for dictionaries / mappings, None values are not allowed, '{}' is interpreted as an empty dictionary

LoG:

# Because of resampling to (3, 3, 3), the use of sigmas < 3 mm is not recommended.

sigma: [3.0, 5.0]

Wavelet: {}

# Featureclasses, from which features must be calculated. If a featureclass is not mentioned, no features are calculated

# for that class. Otherwise, the specified features are calculated, or, if none are specified, all are calculated (excluding redundant/deprecated features).

featureClass:

# redundant Compactness 1, Compactness 2 an Spherical Disproportion features are disabled by default, they can be

# enabled by specifying individual feature names (as is done for glcm) and including them in the list.

shape:

firstorder: [] # specifying an empty list has the same effect as specifying nothing.

glcm: # Disable SumAverage by specifying all other GLCM features available

- 'Autocorrelation'

- 'JointAverage'

- 'ClusterProminence'

- 'ClusterShade'

- 'ClusterTendency'

- 'Contrast'

- 'Correlation'

- 'DifferenceAverage'

- 'DifferenceEntropy'

- 'DifferenceVariance'

- 'JointEnergy'

- 'JointEntropy'

- 'Imc1'

- 'Imc2'

- 'Idm'

- 'Idmn'

- 'Id'

- 'Idn'

- 'InverseVariance'

- 'MaximumProbability'

- 'SumEntropy'

- 'SumSquares'

glrlm: # for lists none values are allowed, in this case, all features are enabled

glszm:

gldm: # contains deprecated features, but as no individual features are specified, the deprecated features are not enabled
