# Supplementary material for: Development and validation of an ultrasound-based radiomics nomogram for predicting the luminal from non-luminal type in patients with breast carcinoma
Source: Front Oncol. 2022 Nov 28;12:993466. doi: 10.3389/fonc.2022.993466 (PMC9749858; doi:10.3389/fonc.2022.993466)
Supplement: Supplementary file 2 [file DataSheet_2.docx]

**Rad-score** =

1.66528+

-0.53842*original_firstorder_Kurtosis+ 0.62226*original_glszm_SmallAreaEmphasis+ 0.59434*original_glszm_SmallAreaEmphasis +

-0.11826*wavelet.LLH_firstorder_Kurtosis+ -0.82805*wavelet.LHL_glszm_SizeZoneNonUniformity+ -0.44651*wavelet.LHH_glszm_SizeZoneNonUniformityNormalized+ 0.03531*wavelet.LHH_glszm_SmallAreaLowGrayLevelEmphasis+ -0.53810*wavelet.HLL_firstorder_Entropy+ -0.19865*wavelet.HHL_glszm_LowGrayLevelZoneEmphasis+ -0.72292*wavelet.HHL_gldm_DependenceNonUniformityNormalized+ 0.12567*wavelet.HHH_firstorder_Energy
